# Genetic regulation and variation of fetal plasma metabolome in the context of sex, paternal breeds and variable fetal weight

**DOI:** 10.1098/rsob.240285

**Published:** 2025-03-05

**Authors:** Siriluck Ponsuksili, Eduard Murani, Beate Fuchs, Christina E. Galuska, Henry Reyer, Muhammad Arsalan Iqbal, Shuaichen Li, Michael Oster, Klaus Wimmers

**Affiliations:** ^1^ Genetics and Genomics, Research Institute for Farm Animal Biology (FBN), Dummerstorf 18196, Germany; ^2^ Core Facility Metabolomics, Research Institute for Farm Animal Biology (FBN), Dummerstorf 18196, Germany; ^3^ Faculty of Agricultural and Environmental Sciences, University of Rostock, Justus-von-Liebig-Weg 6b, Rostock 18059, Germany

**Keywords:** IUGR, plasma, metabolome, fetal weight, pig

## Abstract

Metabolic processes in fetuses can significantly influence piglet weight at birth. Understanding the genetic determinants of systemic metabolism is crucial for uncovering how genetic and molecular pathways impact biological mechanisms, particularly during the fetal phase. We present data on 1112 plasma metabolites using untargeted ultra-high performance liquid chromatography-tandem mass spectrometry methods, of 260 backcross (BC) fetuses from two sires’ breeds at 63 days post-conception. Eight chemical superclasses have been identified, with lipids accounting for the majority of metabolites. Genomic heritability (h²) was estimated for each metabolite, revealing that 50% had h² values below 0.2, with a higher average in the amino acid class compared with the lipid. We annotated 448 significant metabolite quantitative trait loci associated with 10 metabolites, primarily lipids, indicating strong genetic regulation. Additionally, metabolite associations with sex, fetal weight and sire’s breed were explored, revealing significant associations for 354 metabolites. Fetal weight influenced the largest number of metabolites, particularly glycerophospholipids and sphingolipids, emphasizing the genetic and metabolic complexity underlying fetal development. These findings enhance our understanding of the genetic regulation of metabolite levels and their associations with key phenotypic traits in fetuses, providing insights into metabolic pathways, potential biomarkers and serving as a baseline dataset for metabolomics studies of fetuses.

**Figure:**
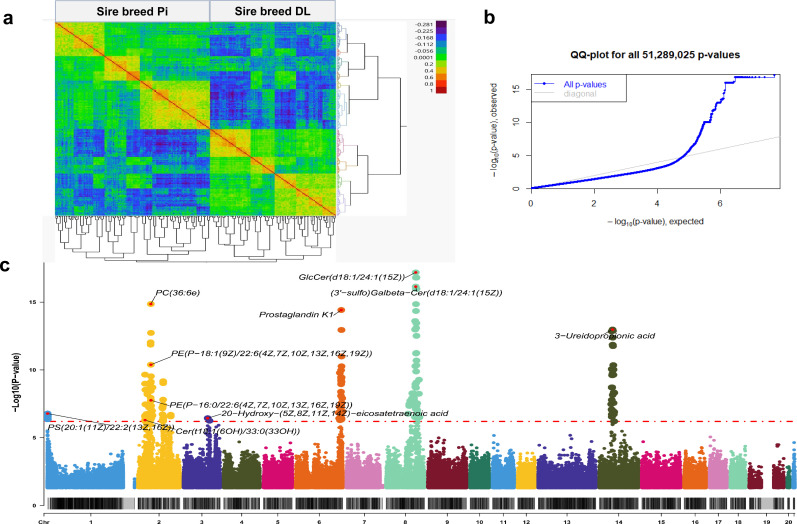

## 1. Introduction

Intrauterine growth restriction (IUGR) occurs when the fetus receives insufficient nutrients and oxygen, often due to maternal malnutrition or placental insufficiency. This results in a fetal weight that is at least 2 s.d. below the average for its gestational age [1,2]. It has been estimated that approximately 15–20% of piglets in each litter are affected by growth restriction in the uterine horn, which results in a low birth weight for the piglets [1]. Metabolites are small molecules produced by metabolic reactions, either as intermediates or end products. The composition or levels of these metabolites are influenced by factors, such as genetics, diet, lifestyle and health conditions [3,4]. A previous study investigated the changes in liver metabolites in discordant siblings with extreme fetal weights and revealed that the changes involved various lipid metabolic pathways, especially in the category of sphingolipids and phospholipids [5]. Some studies have identified metabolites associated with delayed growth (e.g. proline and myo-inositol) and, thus, with lower maturity [6]. Gaining insight into the causal role of plasma metabolites during the fetal period may identify key intervention points for studying the IUGR process. A potential approach to evaluating the role of metabolites in fetal outcomes is through genetic analysis. Indeed, Hagenbeek *et al*. reviewed that approximately 50% of total phenotypic differences in metabolite levels are due to genetic variance [7]. They confirmed the heritability estimates of several hundred blood metabolites, particularly emphasizing that unsaturated phosphatidylcholines have higher heritability than their saturated counterparts [7]. Genome-wide association studies (GWASs) in humans coupled with metabolic profiling platforms have successfully identified many loci associated with metabolic traits [8,9]. Our previous genome-wide association analyses revealed metabolite quantitative trait loci (mQTL) of trans-4-hydroxy-l-proline, being strongly correlated with plasma creatinine levels in pigs [10]. Moreover, mGWAS analysis with differences in feed efficiency provided significant metabolites as potential genetic or biomarkers for feed efficiency [11]. Other studies have sought to identify genomic regions that influence the genetic variance of metabolites, which are genetically linked to resilience and production traits [12].

In this study, a genome-wide association analysis and genomic heritability estimation have been performed on 1112 non-targeted metabolites from 260 mid-term fetuses (day 63 post-conception; dpc) derived from a backcross (BC) of F1 sows (German Landrace (DL) × Pietrain (Pi)) and DL or Pi sires. Blood plasma was chosen at this specific time point because it coincides with critical developmental milestones in the pig fetus, including significant liver maturation and myogenesis around 63 dpc, and paralleling with our previous studies in F2 fetuses [5,13–15]. Analysing plasma at this stage provides a comprehensive screening of systemic metabolic changes, and offers an opportunity to identify biomarkers relevant to fetal health and development. The influence of the sire’s breed on these metabolites with regard to BC breeding is analysed. Furthermore, the relationship between the co-expression of metabolite groups and fetal body weight is investigated.

## 2. Results

**Figure 1.**
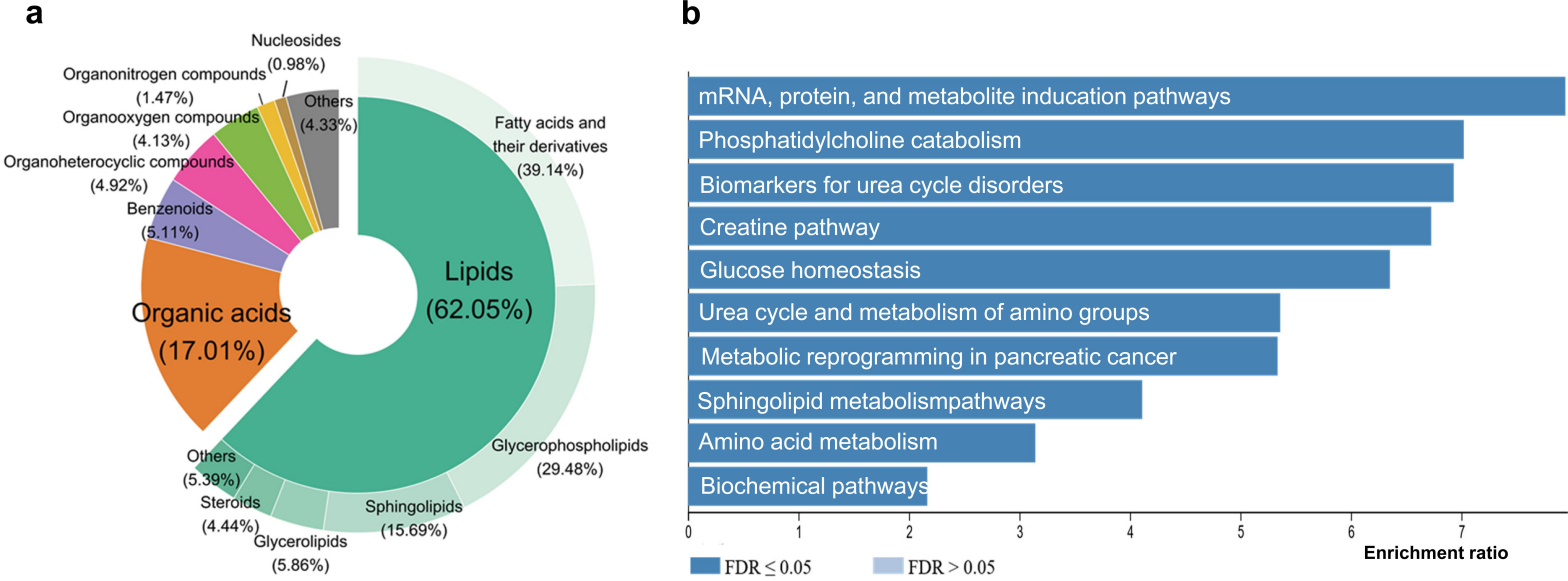
Overview of blood plasma metabolome dataset. (a) Chemical composition of pig fetal blood plasma metabolome categorized into various classes. (b) Top 10 mapped pathways of metabolites identified using the Human Metabolome Database (HMDB; http://www.hmdb.ca) and KEGG Database. HMDB IDs were subjected to pathway analysis using [17].

A total of 1112 metabolites were identified using untargeted methods from extractions with methanol and isopropanol. Both extracts were analysed in positive and negative ionization modes through reverse-phase ultra-high performance liquid chromatography-tandem mass spectrometry (UHPLC-MS/MS). Specifically, 119 metabolites were derived from the methanol extraction in negative ionization mode (MeOH_neg), 632 from the methanol extraction in positive ionization mode (MeOH_pos), 67 from the isopropanol extraction in negative ionization mode (Lip_neg) and 294 from the isopropanol extract in positive ionization mode (Lip_pos). Out of 1112 metabolites (electronic supplementary material, table S1), 1017 were manually classified into eight chemical superclasses, following a system similar to the ClassyFire classification [16]. The majority fall under lipids, accounting for 62.0% (631 of 1017). Under the category of lipids, 39.1% are classified as fatty acids, fatty acyls and their derivatives (247 of 631). Glycerophospholipids make up 29.5% (186 of 631), and this group includes glycerophosphoglycerols (PG), glycerophosphoinositols (PI), glycerophosphothreonines (PT), lysophosphatidylcholines (LPC), glycerophosphoethanolamines (PE), glycerophosphoserines (PS) and glycerophosphocholines (PC). Sphingolipids constitute 15.7% (99 of 631) and include ceramides (Cers), glycosphingolipids (GlcCer/GalCer) and phosphosphingolipids (SM). Glycerolipids account for 5.9% (37 of 631), including triradylglycerols (TG) and diradylglycerols (DG). Steroids make up 4.4% (28 of 631), while prenol lipids represent 3.0% (19 of 631). Finally, 0.8% are ceramides (5 of 631). Most organic acids/compounds in this study, including amino acids, modified amino acids, hydroxyl acids, keto acids and organic phosphoric acids, constitute 17.0% (173 of 1017). The remaining compounds are categorized as follows: benzenoids at 5.1% (52 of 1017), organoheterocyclic compounds at 4.9% (50 of 1017), organooxygen compounds at 4.0% (41 of 1017), organonitrogen compounds at 1.5% (15 of 1017), nucleosides at 1.9% (19 of 1017), and others at 3.2% (33 of 1017) (figure 1*a*).

The majority of all fetal plasma metabolites consist of complex lipids and organic acids, which are only minimally represented in standard biochemical pathways. Therefore, we queried the metabolites using the Human Metabolome Database (HMDB), resulting in 520 of 1112 metabolites being annotated. These HMDB IDs were subjected to pathway analysis using WebGestalt 2024, which now supports metabolomics [17]. For the selected reference set, WikiPathways was used to provide most pathway-based metabolite sets (electronic supplementary material, table S2). The top 10 pathways are illustrated in figure 1*b*, indicating important pathway modules to interpret metabolic changes in the fetus plasma, including biochemical pathways, biomarkers for urea cycle disorders, urea cycle and metabolism of amino groups, amino acid metabolism, phosphatidylcholine catabolism, sphingolipid metabolism, glucose homeostasis and the creatine pathway.

### 2.1. Genomic heritability and metabolite quantitative trait loci

**Figure 2.**
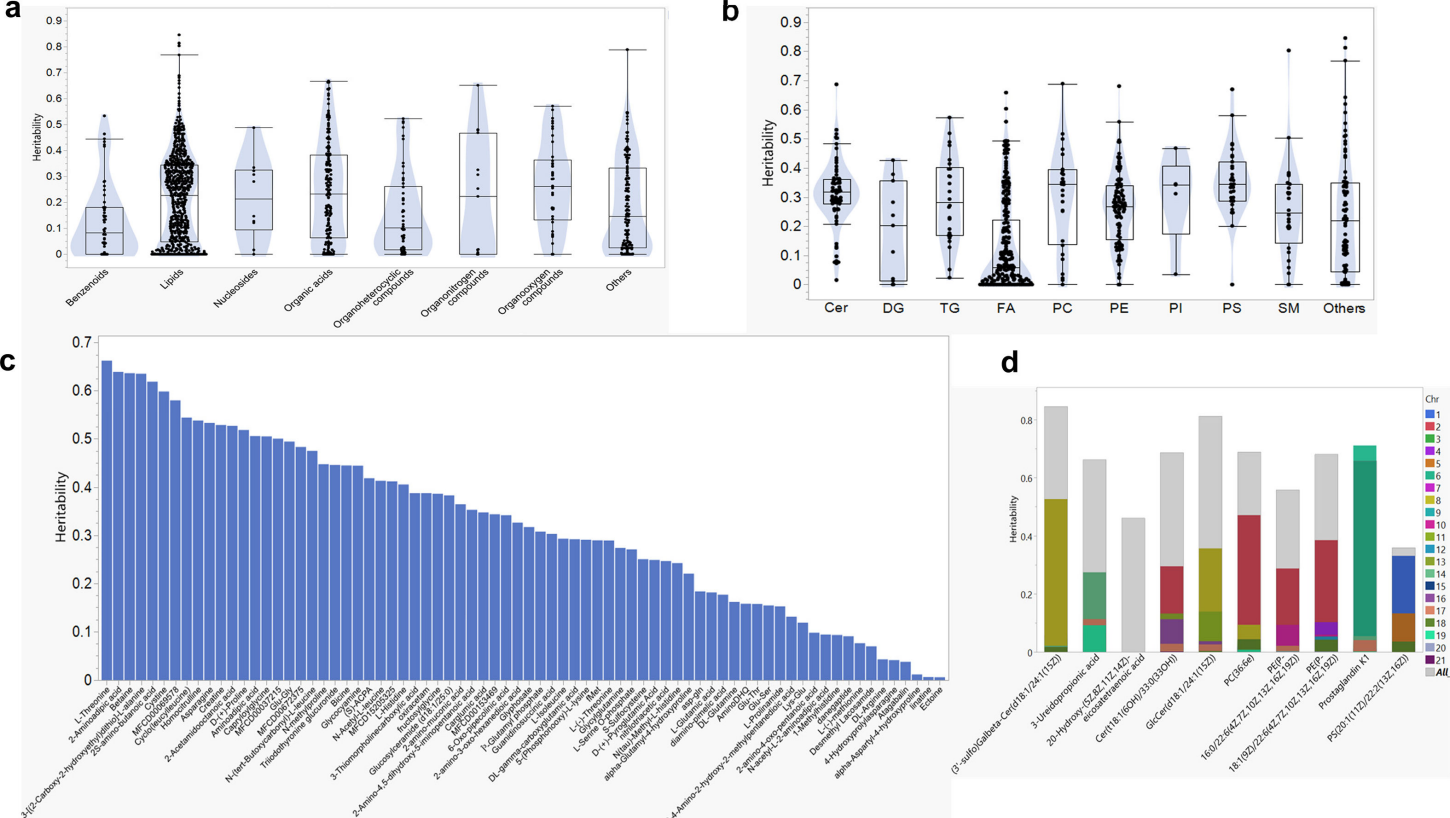
Genomic heritability estimation of metabolites. (a) Box plot showing the heritability of metabolites across eight chemical superclasses. (b) Box plot displaying the heritability of metabolites within the lipid superclass, including ceramides (Cers), triradylglycerols (TG), diradylglycerols (DG), fatty acids and their derivatives (FA), glycerophosphocholines (PC), glycerophosphoethanolamines (PE), glycerophosphoinositols (PI), glycerophosphoserines (PS), phosphosphingolipids (SM) and others. (c) Each column bar indicate heritability of metabolites classified within the amino acids group. (d) Heritability of metabolites with significant mQTLs. The colours in each column represent the proportion of heritability attributed to each chromosome.

To evaluate the genetic impact on the variance of metabolite levels among individuals, we estimated the genomic heritability (h²) of these levels using the genomic-relatedness matrix restricted maximum likelihood (GREML) method. We found that 50% (557 of 1112 metabolites) of the features had h² estimates lower than 0.2, while less than 1% had h² estimates higher than 0.7 (electronic supplementary material, table S3). The genomic heritability across each cluster is shown in figure 2*a*, with the majority of identified features falling into the lipid category. The mean ± s.d. heritability for a subset of features within the lipid category was approximately 0.21 ± 0.17 (figure 2*b*), compared to 0.31 ± 0.18 for amino acids (figure 2*c*). Higher h² values, ranging from 0.35 to 0.84, were observed for metabolites identified as significant mQTLs (figure 2*d*).

**Figure 3.**
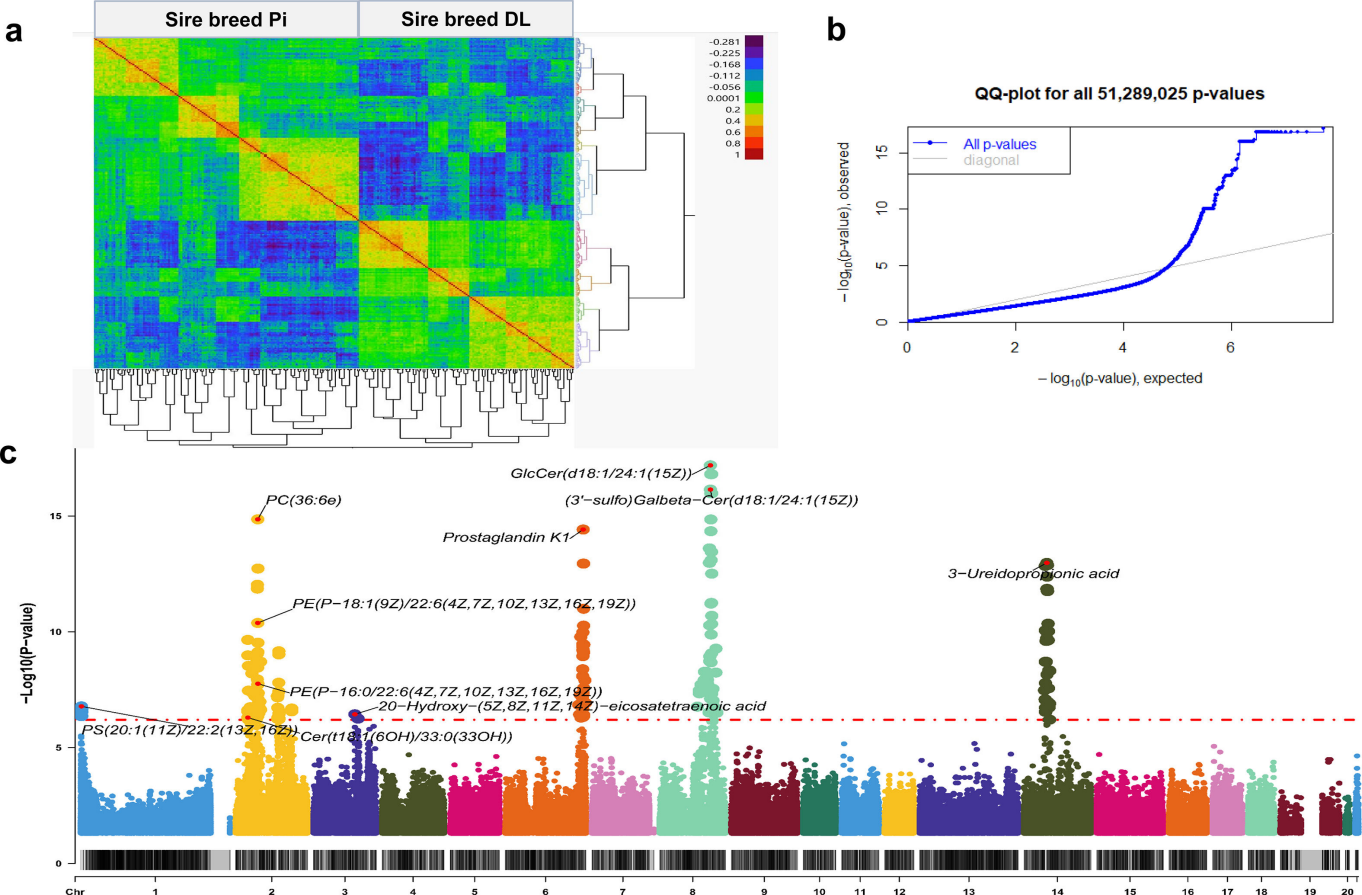
mQTL for 1093 blood plasma metabolites. (a) Hierarchical clustering of the SNPs reveals the genetic relationship among samples based on sire breeds. The colours indicate the degree of relatedness (identity by descent). (b) QQ-plot of all *p*-values. (c) Manhattan plot of mQTLs study of metabolite abundances in blood plasma samples from 63 dpc fetuses (*n* = 260) of a BC population. The names of the top 10 metabolites with significant loci are shown. The horizontal dotted line represents the significance threshold of FDR < 0.1 (*y*-axis, -log10 *p*-values), while the *x*-axis shows the chromosomal number and position.

Genotypes obtained from 260 fetal samples were used to analyse the genetic regulation of metabolites (mQTL), including 1112 plasma metabolites. The genetic relationship of the 260 samples sired by DL and Pi is shown in (figure 3*a*). A GWAS covering 46 925 single nucleotide polymorphism (SNP) genotypes and 1093 metabolites, as demonstrated in the QQ-plot for all 51 289025 *p*-values (figure 3*b*), revealed 448 significant mQTLs corresponding to 10 metabolites and 352 SNPs at a threshold of false discovery rate (FDR) < 0.1 (electronic supplementary material, table S4; figure 3*c*). Nine of these 10 metabolites belong to the lipid category including PS, PE, PC, GlcCer, GalCer, FA and Cer, while only ureidopropionic acid, which belongs to organic acids category, is an exception (table 1). Ureidopropionic acid, a urea derivative of beta-alanine, has an mQTL located on SSC14 (44-54 Mb). The strongest association was for GlcCer(d18:1/24:1(15Z)), which belongs to the glycosphingolipid category, with SNP markers at position 106 Mb on chromosome 8 (*p* = 6.4 × 10^−18^). The metabolite (3′-sulfo)Galbeta-Cer(d18:1/24:1(15Z)), which is a glycosphingolipid containing a sulfate group, has an mQTL located on SSC8 at 79−118 Mb. This region covers many genes, including the peak region mapping at *LOC100512003, CAMK2D* and *ANK2*. 20-Hydroxy-(5Z,8Z,11Z,14Z)-eicosatetraenoic acid belongs to the category of eicosanoids, which are signalling molecules derived from 20-carbon polyunsaturated fatty acids (PUFAs), primarily arachidonic acid. The mQTL of 20-hydroxy-(5Z,8Z,11Z,14Z)-eicosatetraenoic acid is located on SSC3 at 87−94 Mb, overlapping with *ATP6V1E2* and *LOC110260021*. Prostaglandin K1 also belongs to the same category of eicosanoids, with its mQTL located on SSC6 at 155−169 Mb.

**Table 1.** Results of metabolite quantitative trait loci (mQTL) associated with 10 metabolites, including their heritability estimates, analysed using single-marker analyses (generalized linear mixed model) in BC fetuses (*n* = 260).

ID	metabolites	class	heritability	*p*‐value	FDR	SSC (Mb)
lipids-neg−752	(3'-sulfo)Galbeta-Cer(d18:1/24:1(15Z))	sphingolipid	0.85	1.05 × 10^−16^	1.49× 10^−10^	8( 106)
lipids_neg_762	20-hydroxy-(5Z,8Z,11Z,14Z)-eicosatetraenoic acid	fatty acids	0.46	3.65× 10^−07^	4.95× 10^−02^	3 (87)
lipids_neg_789	prostaglandin K1	eicosanoids	0.66	3.82× 10^−15^	5.03× 10^−09^	6 (164)
lipids_neg_808	PS (20:1(11Z)/22:2(13Z,16Z))	glycerophospholipids	0.36	1.66× 10^−07^	2.64× 10^−02^	1 (122)
lipids_pos_1050	PE (P−16:0/22:6(4Z,7Z,10Z,13Z,16Z,19Z))	glycerophospholipids	0.81	1.75× 10^−08^	3.49× 10^−03^	2 (46)
lipids_pos_1054	PE (P−18:1(9Z)/22:6(4Z,7Z,10Z,13Z,16Z,19Z))	glycerophospholipids	0.69	4.14× 10^−11^	2.06× 10^−05^	2 (46)
lipids_pos_918	Cer (t18:1(6OH)/33:0(33OH))	sphingolipids	0.67	5.06× 10^−07^	6.40× 10^−02^	2 (25)
lipids_pos_943	GlcCer (d18:1/24:1(15Z))	sphingolipids	0.81	6.47× 10^−18^	4.43× 10^−11^	8 (106)
lipids_pos_987	PC (36:6e)	glycerophospholipids	0.69	1.39× 10^−15^	1.90× 10^−09^	2 (46)
MeOH_pos_288	3-ureidopropionic acid	organic carbonic acids (ureas)	0.66	1.06× 10^−13^	9.08× 10^−08^	14(48-49)

### 2.2. Metabolites analysis in the context of sex, fetal weight and sires’ breed

**Figure 4.**
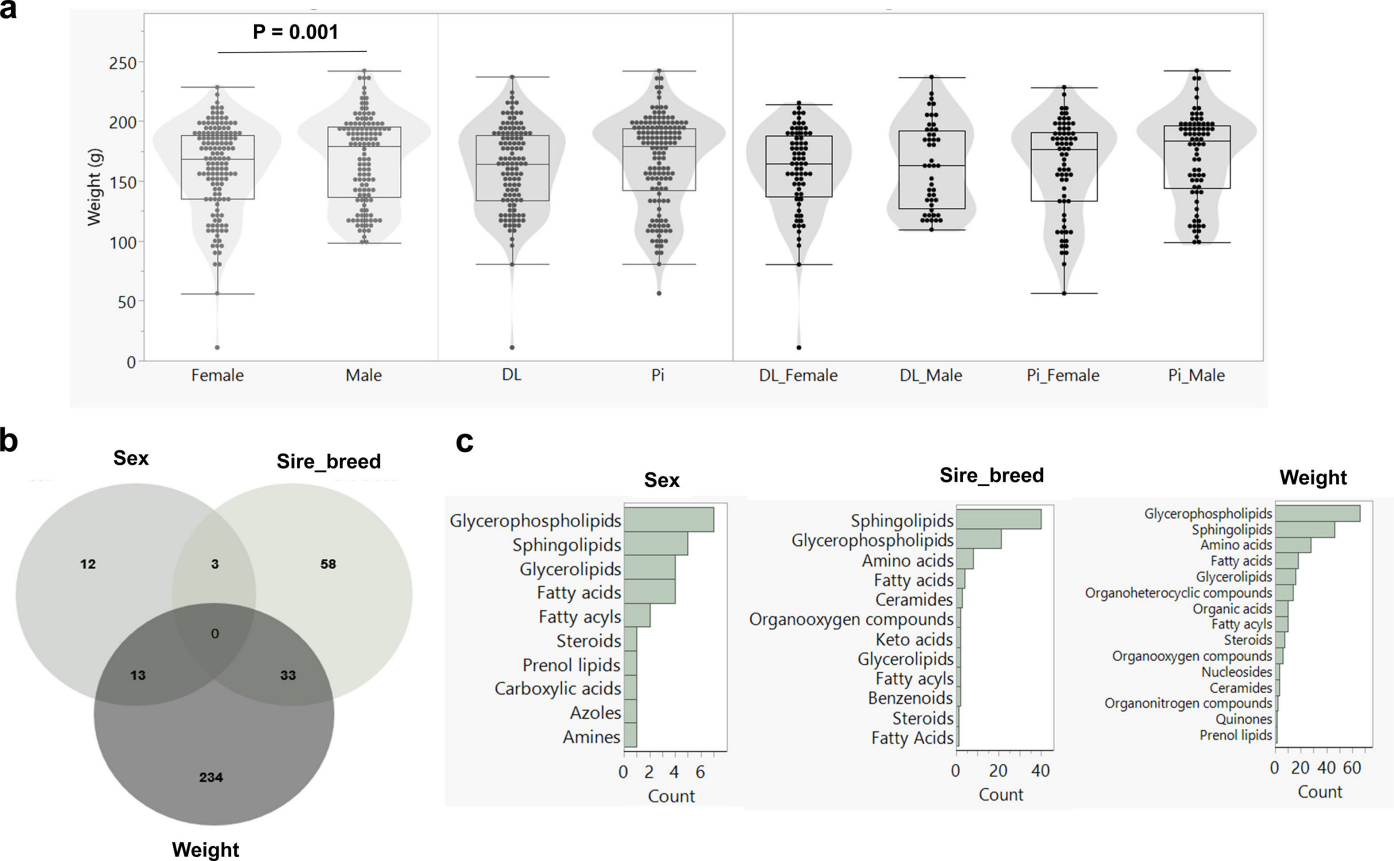
The impact of factors on fetal weight and their blood plasma metabolites. (a) Significant difference in fetal weight at 63 dpc between male (*n* = 121) and female (*n* = 139) fetuses. No significant difference was observed between sire breeds or the interaction between sire breeds and sex. (b) Venn diagram displaying the number of blood plasma metabolites associated with sex, sire breed, and fetal weight. (c) Categories of metabolites associated with sex, sire breed or fetal weight.

Fetus weight data of 260 BC samples (121 males and 139 females) from three DL sires and three Pi sires mated to 22 F1 dams were available and averaged 163 ± 37 g (figure 4*a*). The number of fetuses per dam ranged from 9 to 15. A significant difference in fetal weight at 63 dpc was found between the sexes (*p* = 0.001; figure 4*a*). The average weight of male and female fetuses was 167 ± 36 and 160 ± 37 g, respectively. There was no significant effect of sire’s breed or the interaction of sire’s breed*sex on fetal weight.

In total, 1112 plasma metabolites from fetuses were analysed to assess their associations with sex, fetal weight and sires' breed. Among these, 354 metabolites showed significant associations (FDR < 10%, electronic supplementary material, table S5). Specifically, 28 metabolites were significantly associated with sex, 94 with sires' breed and 280 with fetal weight (figure 4*b*). Here, 24 out of 28 metabolite belong to the class of lipids including fatty acids, glycerolipids, glycerophospholipids, prenol lipids, sphingolipids and steroids (figure 4*c*). About 94 metabolites were associated with the sire’s breed, of which 86 showed higher abundances in fetuses derived from DL sire compared to those of Pi sires. Most of these metabolites belonging to the lipid category, including sphingolipids, glycerophospholipids and fatty acids (figure 4*c*). Fetal weight had the most significant impact, being associated with the largest number of metabolites (figure 4*b*), belonging to glycerophospholipids, sphingolipids and amino acids (figure 4*c*). Additionally, five metabolites were identified as genetically regulated (mQTLs). These included (3′-sulfo) Galbeta-Cer(d18:1/24:1(15Z)) and GlcCer(d18:1/24:1(15Z)), which were influenced by both fetal weight and the sire’s breed. Prostaglandin K1 and PS(20:1(11Z)/22:2(13Z,16Z)) were affected by fetal weight, while Cer(t18:1(6OH)/33:0(33OH)) was influenced by the sire’s breed.

### 2.3. Weighted gene co-expression network analysis

**Figure 5.**
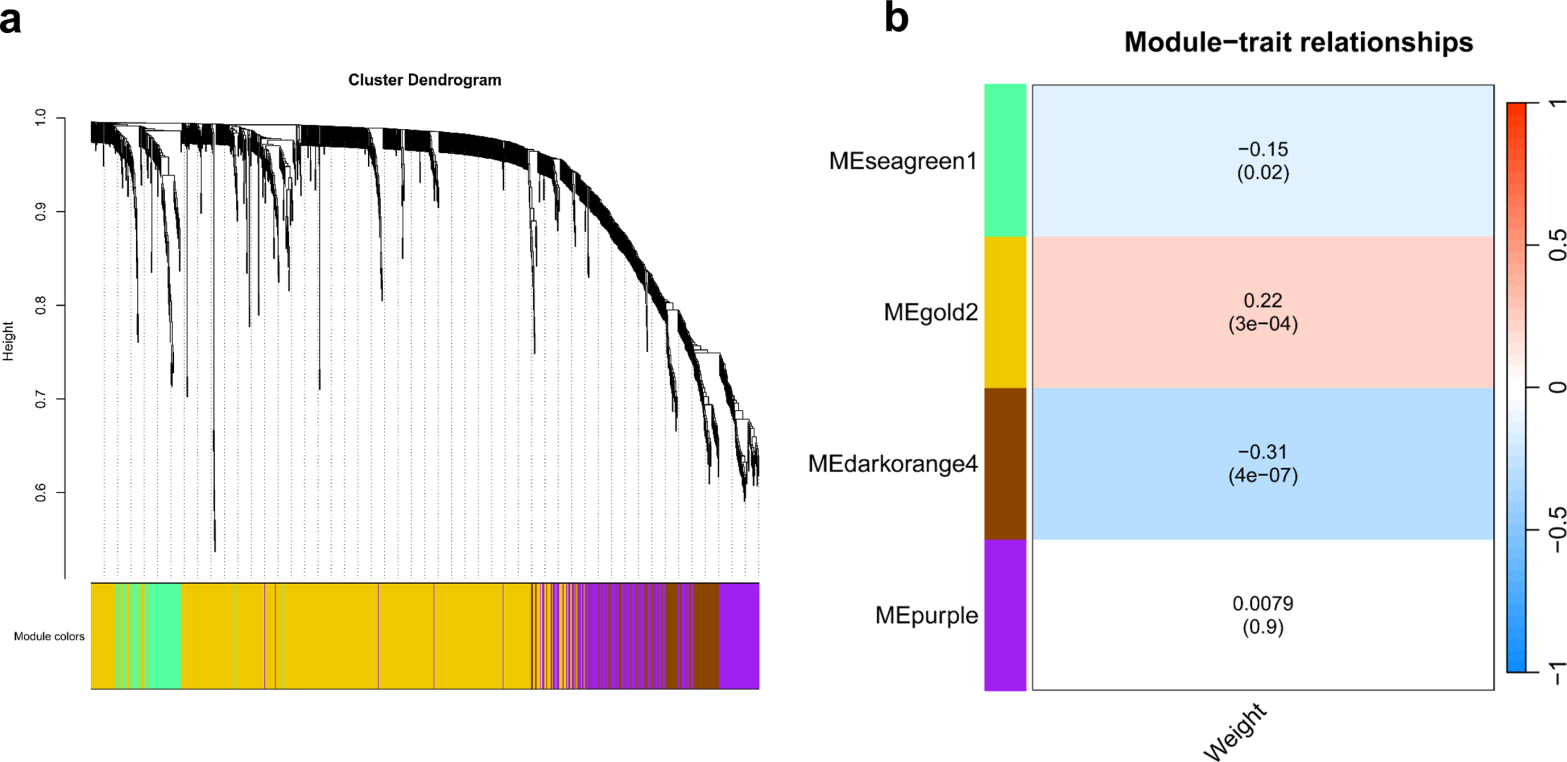
WGCNA of metabolites. (a) The dendrogram shows the hierarchical clustering of metabolites based on topological overlap, used for detecting modules via dynamic tree cutting. (b) The heatmap illustrates the module-trait relationships, with colour gradients representing the strength and direction of correlations. Notably, metabolites in the darkorange4 module are negatively correlated with fetal weight, while those in the gold2 module are positively correlated.

To explore the correlation of metabolite groups with fetus weight, weighted gene co-expression network analysis (WGCNA) was performed. This method clusters metabolites with similar patterns into distinct modules, allowing us to identify potential associations with fetal weight. In total, four metabolite modules were identified (electronic supplementary material, table S6). The correlations between these modules and the fetal weight were evaluated (figure 5, electronic supplementary material, table S6). Our data showed that the module ‘darkorange4’ involving 123 metabolites, was negatively correlated with fetal weight while the module ‘gold2’ representing 667 metabolites was positively correlated with fetal weight. Most of the metabolites in the module ‘darkorange4’ belong to the metabolites extracted with isopropanol. About 178 metabolites from the modules ‘darkorange4’ (87 metabolites) and ‘gold2’ (91 metabolites) overlapped with results of the metabolite analysis using mixed model based on fetal weight. This overlap suggests a consistent relationship between these metabolites and fetal weight, supporting their potential biological significance.

## 3. Discussion

In this study, we utilized an untargeted metabolomics platform to capture a snapshot of thousands of metabolites present in fetal plasma. Factors including weight, sex and breed affect the metabolites levels [18–20]. Metabolomics analyses have been utilized in metabolic phenotyping studies of human populations to determine the most effective strategies for metabolic assessments [21]. In pig husbandry, low birth weight of piglets is an important factor affecting vitality and performance [22]. The results of the metabolome analysis in the umbilical cord serum of piglets with low and normal birth weights indicate a particular difference in amino acid and sphingolipid metabolism [20]. Other studies have shown that lipid metabolism, including the metabolism of phospholipids, sphingolipids and cholesterol, plays a crucial role in signalling pathways that regulate embryo development, implantation and uterine decidualization [23,24]. However, the role of lipid metabolism in fetus development is not yet fully understood. Maternal lipid metabolism supports cell growth, signalling and the development of key structural and functional features of the feto-placental unit [25]. Fatty acids supply essential energy for the fetus, while phospholipids and sphingolipids play key roles as signalling molecules and are crucial for cell membrane formation and tissue development [26]. A relatively low number of mQTLs was observed, which may be due to the small sample size or factors specific to the fetal state. Our findings are consistent with other metabolite QTL studies that report a low number of significant hits despite analysing large metabolite panels [10,27]. In our mQTL analysis, nine of ten annotated metabolites fall into the lipid category, including phospholipids, sphingolipids, glycerophospholipids, fatty acids and prostaglandins. Among these, two sphingolipids [(3'-sulfo)Galbeta-Cer(d18:1/24:1(15Z)) and GlcCer(d18:1/24:1(15Z))] showed the strongest mQTL associations with higher h^2^ more than 0.8, with sire breed and fetal weight being significant factors influencing these metabolites. Additionally, Cer(t18:1(6OH)/33:0(33OH)) is strongly associated with genetic variants on SSC2, with sire breed also impacting this metabolite. The four glycerophospholipids identified PS(20:1(11Z)/22:2(13Z,16Z)), PE(P−16:0/22:6(4Z,7Z,10Z,13Z,16Z,19Z)), PE(P−18:1(9Z)/22:6(4Z,7Z,10Z,13Z,16Z,19Z)) and PC(36:6e)) also highlight the importance of genetic regulation in lipid metabolism. Notably, PS(20:1(11Z)/22:2(13Z,16Z)) as significantly associated with fetal weight, suggesting a potential link between this specific glycerophospholipid and fetal growth and development. Other significant genetic variants regulate metabolites in the fatty acid category, including 20-hydroxy-(5Z,8Z,11Z,14Z)-eicosatetraenoic acid and prostaglandin K1. Prostaglandins (PGs), produced through the hydrolysis of membrane phospholipids, are involved in a wide range of physiological processes, including the regulation of inflammation [28]. Key prostaglandins like *PGE2* and *PGF2α* are crucial for immunomodulation at the maternal–fetal interface and for pregnancy recognition during early stages in pigs [29,30]. While the specific functions of prostaglandin K1 are not yet understood, it is part of the broader prostaglandin family and may be involved in signalling pathways related to inflammation and other prostaglandin-mediated processes. Notably, we found that prostaglandin K1 is highly heritable (h² = 0.66), with significant mQTLs on SSC6 (*p* < 3.8 × 10^−15^), and is associated with fetal weight (*p* < 0.005), highlights prostaglandin K1 as both genetically controlled and biologically relevant in influencing fetal weight, possibly making it a target for future research on fetal growth regulation.

Ureidopropionic acid, also known as N-carbamoyl-β-alanine, has an mQTL located on SSC14 (44–54 Mb). This compound is a substrate for the enzyme β-ureidopropionase (β-UP), which plays a role in converting this intermediate into other products as part of the pyrimidine catabolism [31]. Elevated concentrations of ureidopropionic acid lead to a diagnosis of β-UP deficiency, which was discovered as an inborn error of metabolism or observed in patients with Dravet syndrome [31,32]. β-UP deficiency is a rare autosomal recessive disease caused by mutations in the β-UP gene (*UPB1*) as reported by many studies in human [33–35]. In pig, *UPB1* is located on SSC14 (49.53−49.56 Mb). Our previous study on genetically regulated liver transcripts demonstrated *cis*-regulation of *UPB1* [10,36]. In the current study, the same SNP markers (rs80816587, rs80868709, rs328507444, rs80820789, rs80941022, rs80871817, rs80836220, rs80832510, rs80807464, rs80990511 and rs80921375) that regulate ureidopropionic acid were also found to regulate the transcript of *UPB1*. This study suggests that genetically regulated metabolites and associated transcripts could serve as potential biomarkers for fetal metabolic disorders. In addition, the identified mQTLs were linked to 10 metabolites with higher heritability. While mQTL signals indicate specific genetic loci, heritability reflects the overall genetic influence on trait variation.

Fetus and placental growth in pigs is influenced by various factors, including genetic, epigenetic, and environmental elements [37]. The sphingolipid pathway involves not only components of the cell membrane but also bioactive lipids that serve as signalling molecules in cellular processes, essential for maintaining a normal pregnancy [38,39]. Our previous study demonstrated that IUGR predominantly affects lipid metabolism in the liver, particularly sphingolipids and phospholipids [5]. This finding aligns with our current study in fetal plasma, where we observed that lipid metabolism, specifically sphingolipids and phospholipids, is also influenced by fetal weight. Additionally, our previous research reported sexually dimorphic muscle transcripts, particularly those located on sex chromosomes, occurring at the fetal stage [15]. Notably, we found that sex and fetal weight were significantly associated with these TG metabolites. Triacylglycerol biosynthesis, which involves the storage of excess dietary energy as lipid droplets in adipose tissue, is crucial for energy homeostasis [40]. Furthermore, the differences in lipid profiles between male and female fetuses could be attributed to hormonal influences and genetic factors that regulate lipid metabolism differently in each sex [41]. Aside from lipid metabolism, amino acids also impact fetal weight [20]. In sheep, research suggests that the umbilical uptake of amino acids is preferentially transferred to the fetus during late gestation [42]. In our study, approximately 30 amino acids were detected, most of which are part of the ‘gold2’ module, which is positively correlated with fetal weight. In contrast, most metabolites in the ‘darkorange4’ module, which belong to lipids and their derivatives, are negatively correlated with fetal weight. Most studies have reported on maternal effects on fetal metabolism, as shown in our previous study on dam effect on fetal transcripts [15,43]. In this study, the focus on paternally inherited genomes from BC of F1 dams with DL or Pi sires revealed that a considerable number of lipid-related metabolites were significantly influenced by sire breeds. Fetuses from DL sires, which are genetically predisposed to store more fat, exhibited higher levels of lipid metabolites. This breed-specific trait likely drives lipid synthesis and storage in DL-sired fetuses, contrasting with the leaner, more muscular characteristics of Pi-sired fetuses. The difference in metabolic focus reflects the distinct biological roles of fat and muscle development between these breeds.

Overall, these findings underscore the complex interplay between genetics, lipid metabolism, amino acids and fetal development, with specific metabolites being closely tied to genetic markers and physiological traits such as breed and fetal weight. This insight could be valuable for understanding the genetic regulation of lipid metabolism in pigs and its implications for fetal development.

## 4. Methods

### 4.1. Animals and sample collection

Our previous studies focused on F2 fetuses from a cross between F1 German Landrace (DL) and Pietrain (Pi) pigs following the same breeding plan and study design [5,15,44,45]. In this study, we focus on a BC strategy by using 22 F1 (German Landrace (DL) × Pietrain (Pi)) sows crossed with 3 DL sires and 3 Pi sires. A total of 270 BC fetuses were obtained at day 63 post-conception. Individual fetal weights were recorded. Liver tissue was immediately snap-frozen in liquid nitrogen after collection and then stored at −80°C for DNA isolation. The fetal blood samples were taken from the umbilical cord after it had been cut and collected in 10 ml EDTA tubes (Sarstedt) for further analysis. These animals were not exposed to any experimental treatment prior to slaughter. The protocols for animal care and tissue collection were reviewed and approved by the Animal Care Committee of the Research Institute for Farm Animal Biology (FBN). All procedures adhered to national animal welfare regulations and complied with EU directive 86/609/EEC on animal experiments.

### 4.2. Single nucleotide polymorphism genotypes

For genotyping, DNA samples (*n* = 270) underwent amplification, fragmentation, and hybridization to the PorcineSNP60 BeadChip (Illumina Inc., San Diego, CA, USA), which includes 62 163 locus-specific 50-mers. Single-base extension of the captured oligonucleotides, labelled in the process, was detected by an Illumina iScan. The resulting images were transformed into intensity data, which were then normalized and processed for genotype calling using GenomeStudio software (version 2.0) (Illumina Inc.). Samples with call rates below 99%, as well as SNP markers with a minor allele frequency of less than 5%, were excluded from the analysis. The overall average call rate was 99.8% ± 0.2. After filtering, 46 925 SNPs from 260 samples remained for subsequent GWAS using a single-marker analysis approach. The 60K chip markers were aligned to the Sus Scrofa 11.1 porcine reference genome.

### 4.3. Metabolomic analysis

Plasma samples are analysed according to protocols established at the FBN Core Facility Metabolomics [46–48]. Briefly, blood samples were collected, centrifuged, and stored at −80°C until extraction. Blood plasma samples were extracted using methanol and isopropanol, respectively, and both extracts were analysed in both positive and negative ionization modes via reverse-phase ultra-high performance liquid chromatography-tandem mass spectrometry (UHPLC-MS/MS) (Vanquish UHPLC system with heated electrospray ionization (HESI) QExactive Plus Orbitrap mass spectrometer; Thermo Scientific, Waltham, USA). Metabolite annotation and relative quantification were carried out using Compound Discoverer 3.2 Software (Thermo Scientific, Waltham, MA, USA). The metabolomics data were further processed by applying the range (IQR) filter, log-transformed, mean-centred, and Pareto scaled (divided by the square root of the standard deviation of each variable) using MetaboAnalyst 4.0 [49]. In total, 1112 metabolites were selected for further analysis. Metabolite annotation was conducted using the HMDB (http://www.hmdb.ca) and the KEGG Database by matching molecular weight, mass-to-charge (m/z) values, retention times, and ion modes. These HMDB IDs were then used for pathway analysis via [17].

### 4.4. Genomic heritability estimation

Genomic heritability represents the proportion of genetic variance explained by SNPs to the phenotypic variance (i.e. metabolites), and was calculated using JMP Genomics (JMP Genomics 10, version 17; SAS Institute, Cary, NC). The genetic influence on traits was estimated based on the SNP data matrix rather than a formal experimental design [50]. The polygenic effect was included in the model employing the genomic relationship matrix using SNP-level variation and this estimated genetic similarity is compared to phenotypic similarity, resulting in heritability estimates. To calculate the heritability of plasma metabolites, sex was included as a fixed effect, with weight used as a covariate. These heritability estimates were obtained using a mixed linear model implemented in JMP Genomics.

### 4.5. Data pre-processing and metabolite quantitative trait loci of metabolome

Following quality control and filtering, 1112 metabolites underwent pre-processing to account for systemic effects. Adjustments were made using mixed-model analyses of variance in SAS. A genetic similarity matrix, calculated as identity by descent for each pair (k-matrix), was included as a random effect. To control for population stratification, the top principal components explaining more than 1% of the variation were included as covariates, with a total of 10 principal components considered. Additionally, sex was treated as a fixed effect in the analysis. The residuals of 1093 metabolites after correction were retained for further analysis. The R package MatrixeQTL was used to test the association between each SNP and the residuals of metabolome abundances, using a least squares model to account for genotype effects [51]. MatrixeQTL conducts individual tests for each metabolite-SNP pair and adjusts for multiple comparisons by calculating the false discovery rate (FDR) [52]. Ensembl_Sscrofa_11.1 was used for annotation and localization of SNP sites.

### 4.6. Metabolites analysis in the context of sex, fetal weight and breed of sires

To examine the variation in metabolite levels due to sex, fetal weight, and the breeds of BC sires, normalized metabolite data were used as dependent variables in an analysis of variance. A linear model (Proc Mixed procedure, SAS 9.4 software; SAS, Cary, USA) was applied, incorporating the fixed effects of sex, sire breed, and sire (sire breed), with dam as a random effect and fetal weight as a covariate. The Tukey–Kramer post hoc test was used to adjust for multiple comparisons across Type III tests for all effects. Additionally, adjusted *p*-values were calculated using the Benjamini–Hochberg method, with significance set at 0.1 [52].

### 4.7. Weighted gene co-expression network analysis

Residuals of 1093 metabolites (after correction, as described above) were used to construct weighted gene co-expression networks via the blockwise modules function in the WGCNA R package, following previously established methods [53–55]. The WGCNA process involved first computing a Pearson correlation matrix for all genes, followed by generating an adjacency matrix by raising the correlation values to a power, ß. The adjacency matrix was then converted into a topological overlap matrix (TOM), which was used to create a TOM-based dissimilarity matrix for hierarchical clustering. Gene co-expression modules were identified from the hierarchical clustering tree using the dynamic tree cut method. The power ß was selected by inspecting the scale-free topology model fit, with the minimal ß chosen to achieve a coefficient of determination (*R*²) greater than 90%. Modules were subsequently merged based on the dissimilarity of their eigengenes, where eigengenes represent the first principal component of each module and serve as the module’s representative. To identify co-expression modules of metabolites highly correlated with fetal weight, module-trait relationships were determined by correlating the module eigengene with the residuals of fetal weight. These residuals were calculated using the same model as for metabolite data, with the genetic matrix as a random effect, the top 10 principal components as covariates and sex as a fixed effect.

## Data Availability

The data are provided in electronic supplementary material at Figshare [56]. Supplementary material is available online [57].
